# Bulk and single-cell transcriptome profiling reveal the metabolic heterogeneity in gastric cancer

**DOI:** 10.1038/s41598-023-35395-y

**Published:** 2023-05-31

**Authors:** Guoqiang Tao, Xiangyu Wen, Xingxing Wang, Qi Zhou

**Affiliations:** Department of General Surgery, Shanghai Punan Hospital, Pudong New District, Shanghai, China

**Keywords:** Cancer models, Tumour immunology, Cancer metabolism

## Abstract

Metabolic reprogramming has been defined as a key hall mark of human tumors. However, metabolic heterogeneity in gastric cancer has not been elucidated. Here we separated the TCGA-STAD dataset into two metabolic subtypes. The differences between subtypes were elaborated in terms of transcriptomics, genomics, tumor-infiltrating cells, and single-cell resolution. We found that metabolic subtype 1 is predominantly characterized by low metabolism, high immune cell infiltration. Subtype 2 is mainly characterized by high metabolism and low immune cell infiltration. From single-cell resolution, we found that the high metabolism of subtype 2 is dominated by epithelial cells. Not only epithelial cells, but also various immune cells and stromal cells showed high metabolism in subtype 2 and low metabolism in subtype 1. Our study established a classification of gastric cancer metabolic subtypes and explored the differences between subtypes from multiple dimensions, especially the single-cell resolution.

## Introduction

Gastric cancer (GC) is the sixth most common cancer and the third leading cause of cancer-related deaths worldwide^1^. Early screening for GC currently relies on gastroscopy; however, this examination is not yet performed annually in many regions, resulting in many GC patients being diagnosed at later stages. Treatment strategies for GC have mainly relied on clinicopathological assessments of tumors, including surgery, various chemotherapy treatments, and immunotherapies targeting immune checkpoints (ICIs). However, since GC is a heterogeneous disease, these treatments only show efficacy in some patients. Currently, the classification of gastric cancer mainly relies on AJCC staging, Lauren classification, and grade, which have limitations. Therefore, it is necessary to classify tumors according to their intrinsic heterogeneity, determine their relationship with tumor treatment, and optimize tumor treatment strategies accordingly.

Metabolic reprogramming has been considered a key hallmark of human tumors^2,3^, as cancer cells require change in their metabolic processes to meet the demands of their rapidly growing biomass and energy needs^4,5^. Metabolic reprogramming plays a crucial role in various tumor processes, including tumor progression, chemotherapy resistance, immune response and epithelial–mesenchymal transition^6–10^. Identification of distinct metabolic isoforms in cancer can aid in patient selection of investigational metabolic inhibitors and new therapeutic targets^11^. Many previous studies have categorized tumors into different metabolic subtypes^12–15^. However, tumor cells exist in a microenvironment composed of stromal cells such as tumor-associated fibroblasts, immune cells, and endothelial cells. Each cell type plays an active role in tumor cell proliferation, and each has unique metabolic needs to perform specific functions. By employing single-cell sequencing technology, we can understand the metabolic characteristics of various types of cells in tumor tissue at the single-cell resolution.

Here, we have initially divided gastric cancer into two subgroups based on bulk sequencing data. We have explored the differences between subtypes from various perspectives, including metabolic subtype, which showed varying responses to various chemotherapeutic agents and immune checkpoint-targeting therapies. Finally, we have explored the metabolic differences in multiple cell types across subtypes from a single-cell dimension.

## Materials and methods

### Data acquisition and processing

We systematically searched publicly available gene expression datasets for GC. Samples without complete prognosis information were removed from further evaluation. In total, 9 datasets from the Gene-Expression Omnibus (GEO; https://www.ncbi.nlm.nih.gov/gds/) (GEO: GSE62254^16^, GSE15459^17^, GSE57303^18^, GSE34942^19^, GSE84437^20^, GSE26942^21^, GSE29272^22^, GSE28541^21^ and GSE13861^23^) and one RNA-sequencing dataset (TCGA-STAD) from The Cancer Genome Atlas (TCGA; https://portal.gdc.cancer.gov/) were found. Because of the large number of datasets on the GPL570 platform, four datasets (GSE62254, GSE15459, GSE57303 and GSE34942) from the GPL570 platform were merged as one dataset named as GPL570 meta-dataset using “oligo” package in R^24^. We used the oligo package in R software for quality control analysis and the “ComBat” function in R to remove the batch effect (Fig. S1)^25^. GSE29272, GSE28541 and GSE13861 were removed due to small sample size. All microarray data included in our study were log2 transformed. Data files of counts expression of TCGA-STAD and clinical data were downloaded by using “TCGAbiolinks” package in R^26^. The data downloaded from TCGA were converted into transcripts per million (TPM) value. TCGA-STAD somatic mutation and DNA methylation profiles with illumina human methylation 450 platform were downloaded using the package “TCGAbiolinks” in R. Somatic mutation data were analysed using R package “maftools”^27^. The chi-square test was used to assess the mutational difference between the two groups. Methylation profiles were analysed using R package “ChAMP”^28^. The beta values were calculated to assess the methylation level of each CpG site in each sample. It is generally considered that β value greater than 0.6 is fully methylated, 0.2–0.6 is partially methylated, and less than 0.2 is completely unmethylated. For differentially methylated probes (DMPs) analysis, we first removed CpG sites that were both fully methylated and fully unmethylated in both clusters. The |diffBeta| was set as 0.15.

Single cell-seq data from GSE183904 were selected for further analysis^29^. This is the largest number size study to date for single-cell sequencing of gastric cancer. Due to the large sample size, the working upper limit of our equipment was exceeded. Therefore, we divided gastric cancer samples into three subgroups according to Lauren classification, and randomly selected one third of the samples from each group, and finally obtained 8 gastric cancer single-cell sequencing samples, and subsequent single-cell analysis was based on these 8 samples. Each sample was considered for genes/features shared by three or more cells, and cells showing 300 or more features. Cells with mitochondrial RNA percentages of > 20 were filtered out. We use the “DoubletFinder” package to remove the "doublets cell"^30^. Tumor specimens also inevitably contain normal epithelial cells. So, “CopyKAT” package was used to predict malignant cells in epithelial cells^31^.

### Metabolic subgroup classification

Metabolite and protein interactions profile was obtained from a previous study^12^. After processing the profile, we input it into Cytoscape software (version 3.6.1) to extract the proteins with more than 5°^32^ (details in Table S1). In the previous step, we obtained 1202 metabolism-related genes. In order to better classify the malignant metabolic characteristics of tumors, we extracted the metabolic genes related to prognosis by “survival” package (P < 0.05). Consensus clustering with 1000 iterations and resampling of 80% was performed based on the expression levels of these genes using “ConsensusClusterPlus” package^33^. We use CDF plot and PAC methods to confirm the best K value.

The single-cell sequencing used was not accompanied by bulk sequencing. We can think of bulk sequencing as measuring the total expression of each gene in all cells in a tumor tissue. Therefore, after the quality control of the single-cell expression matrix was completed, the average expression matrix of all cells in each sample was calculated to estimate the bulk-level expression of a single sample. We have built a metabolic subtype classifier in the TCGA-STAD dataset. The TCGA-STAD dataset is randomly divided into training dataset and test dataset according to 7:3. Metabolic classification model was trained based on prognostic-relevant metabolic genes in the training dataset using support vector machines (SVM) algorithm, and validated in the test dataset. Single-cell samples are classified according to the established classification model.

### Pathway enrichment analyses

We download the latest Kyoto Encyclopedia of Genes and Genomes (KEGG) pathway data using R package “KEGGREST” and made the required “gmt” format file^34–36^. We downloaded the GSEA software (version 4.3) from the gene set enrichment analysis (GSEA: http://software.broadinstitute.org/gsea/index.jsp) website. NOM p-value < 0.05 were considered statistically significant. Single sample gene set enrichment analysis (ssGSEA) was used to estimate KEGG pathway enrichment level in a single sample or cell based on “GSVA” package^37^.

### Evaluation of infiltrating immune cells in the TME

The proportions of 22 immune cell types in GC samples were estimated using the CIBERSORT algorithm (https://cibersortx.stanford.edu/) with batch-corrected mode, relative mode and 1000 permutations of b mode^38^. The estimation of stromal and immune cells in tumor tissues was performed by ESTIMATE algorithm^39^. The wilcoxon test was used to find the significantly different immune cells among different groups.

### Metabolic subtype characteristic score construction

According to the consensus clustering, we could successfully classify patients into two clusters. Differentially expressed genes were determined by using the package “DESeq2” package^40^. The significance criteria for determining DEGs were set as a false discovery rate (FDR) < 0.01 and |log2 fold change (FC)|  > 1.0. In methylation analysis, differential expression analysis between cluster 1 and cluster 2 was also taken to take the above parameters. TCGA-STAD, GPL570 meta-dataset, GSE84437 and GSE26942 were Z-score transformed for subsequent analysis. To remove the effect of low-level expressed genes, we removed genes with average TPM value of less than 2 across all samples. Then, LASSO-Cox regression analysis based on the “glmnet” package in R was applied to build an optimal metabolism classification-related gene signature for GC^41^. The metabolic subtype characteristic score of our model for each sample was defined by the relative expression of each gene and its associated Cox coefficient. The optimal cutoff value was confirmed by “maxstat” package. The patients were divided into high-score group and low-score group, and the Kaplan–Meier (KM) method with logrank test was used to further analyze the prognostic differences between the two groups. The prognostic or predictive accuracy of gene signature was assessed using time-dependent receiver operating characteristic (ROC) analysis. The area under the curve (AUC) at different cutoff times was used to measure the accuracy of prognosis or prediction. The model was then validated on three additional independent datasets.

### Additional bioinformatic and statistical analyses

The half maximal inhibitory concentration (IC50) is estimated by R package “pRRophetic”^42^. The Connectivity Map (CMap, https://clue.io/) was used to predict the small candidate molecules based on differentially expressed genes. The TIDE algorithm was used to predict ICB responses (http://tide.dfci.harvard.edu)^43^. All of the above analyses were performed using the R software (version 4.0.2, http://www.rproject.org). Statistical differences not specifically stated were set at P < 0.05.

## Result

### Metabolism-associated genes and subtypes identification

**Figure 1:**
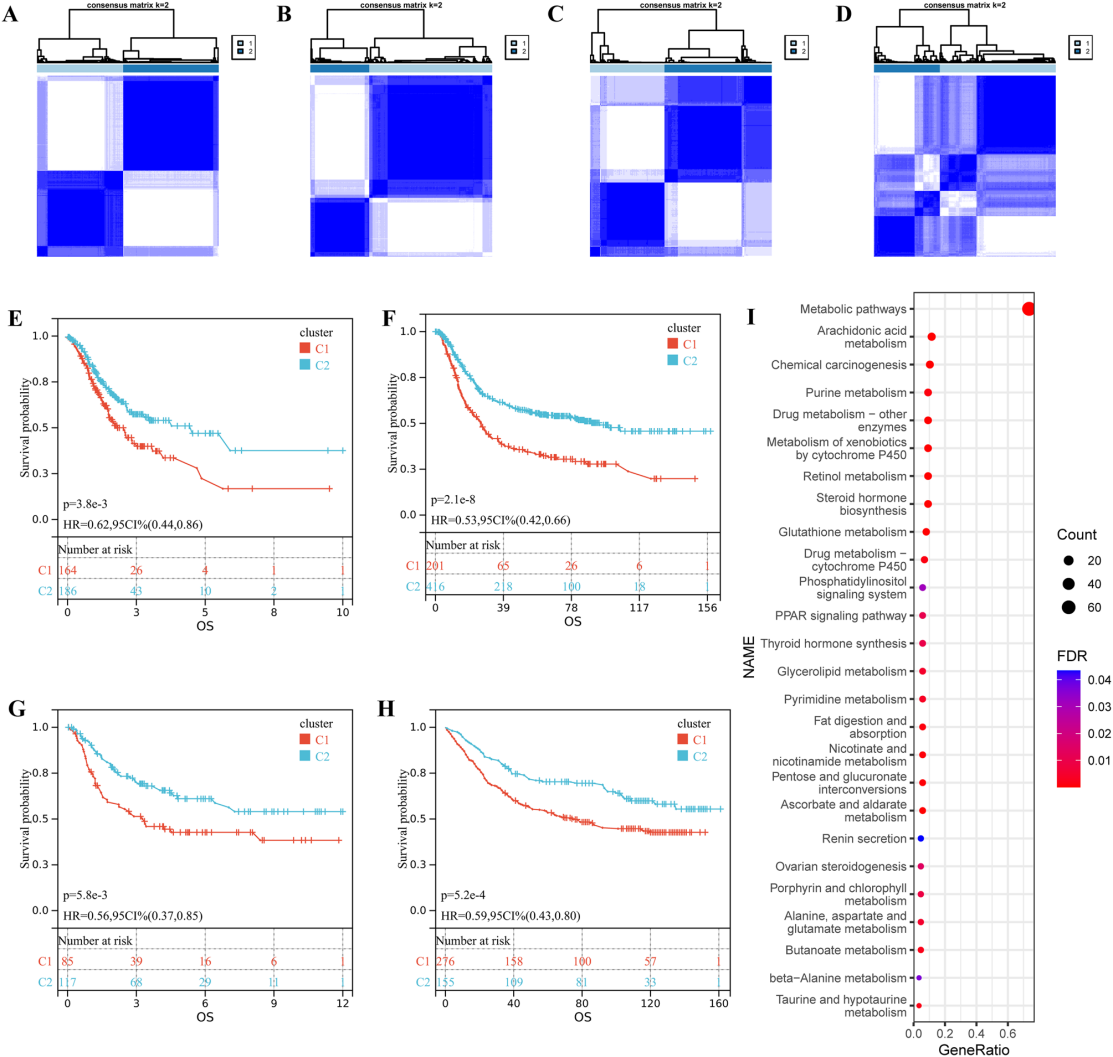
Gastric cancers exhibit metabolic heterogeneity. (**A**–**D**) The consensus matrix shows patients with two metabolic subtypes in TCGA-STAD, GPL570 meta-dataset, GSE26942 and GSE84437. (**E**–**H**) Kaplan–Meier curves for overall survival based on metabolic subtypes (Log-rank test) in TCGA-STAD, GPL570 meta-dataset, GSE26942 and GSE84437. (**I**) KEGG enrichment results of 98 prognosis-related metabolic genes.

98 metabolic related genes (the origin of these genes has been described in the “Materials and methods” section) obtained are significantly enriched in metabolic pathways, including arachidonic acid metabolism, chemical carcinogenesis, drug metabolism—other enzymes and pentose/ glucuronate interconversions, etc. (Fig. 1I). Based on the expression values of these 98 genes, we divided the TCGA-STAD cohort into two clusters, with the optimal k of 2 (Figs. 1A, S2). Our analysis revealed a significant prognostic difference between the two metabolic subtypes (Fig. 1E). Kaplan–Meier analysis showed that patients who were divided into cluster 1 suffered inferior overall survival (OS) (Fig. 1E). Importantly, our clustering result was also verified in the other three cohorts (GPL570 meta-dataset, GSE26942 and GSE84437) (Fig. 1B–D). Furthermore, the difference in prognosis was also observed in these three cohorts (Fig. 1F–H). These results confirmed the metabolic heterogeneity of gastric cancer and its prognostic significance.

### Metabolic classifier-specific gene enrichment pathway of gastric cancers

**Figure 2:**
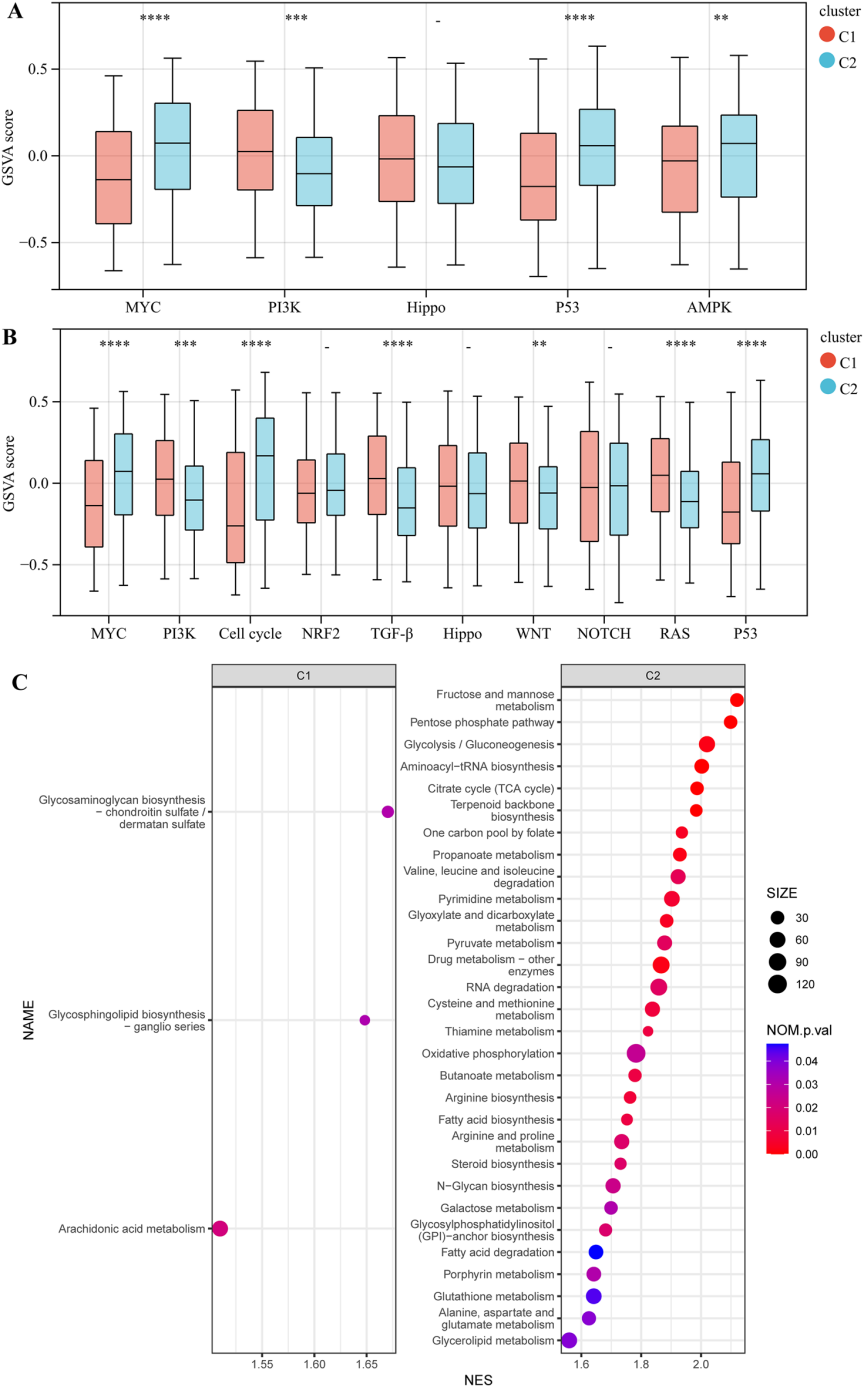
Analysis of signaling pathways between metabolic subtypes. (**A**) Five tumor metabolism-related pathway differences between two metabolic subtypes. (**B**) Ten oncogenic-related pathway differences between two metabolic subtypes. (**C**) Metabolic pathways in two metabolic subtypes using Gene set enrichment analysis. SIZE represents the number of genes in the corresponding gene set. NES represents corrected normalized enrichment score. *P < 0.05, **P < 0.01, ***P < 0.001, ****P < 0.0001.

To understand the metabolic differences and functional differences among various metabolic subtypes, we performed ssGSEA and GSEA analysis. First, the scores of all patient-related pathways were obtained based on the ssGSEA algorithm. We discovered that 4 of the 5 signaling pathways related to tumor metabolism regulation differed between the 2 clusters, which explain the sources of metabolic differences between the 2 clusters (Fig. 2A). Additionally, we observed that 7 of the 10 oncogenic signaling pathways significantly differed between the two clusters, thereby demonstrating the dissimilarities in tumor characteristics between the groups (Fig. 2B).

We then used the GSEA method to analyze the metabolic subtype-specific KEGG pathway. Both subtypes had a considerable number of specific signaling pathways (details in Table S2). Among the metabolic pathways, 3 pathways were significantly enriched in cluster 1, while 30 pathways were enriched in cluster 2 (Fig. 2C). In cluster 1, multiple intercellular communication-related signaling pathways were activated (Table S2). In addition to the activation of a considerable number of metabolic pathways, cluster 2 also showed activation of nucleotide processing and repair-related pathways (Table S2).

### Metabolic classifier-specific mutation, DNA methylation and immune cell infiltration characteristics of gastric cancers

**Figure 3:**
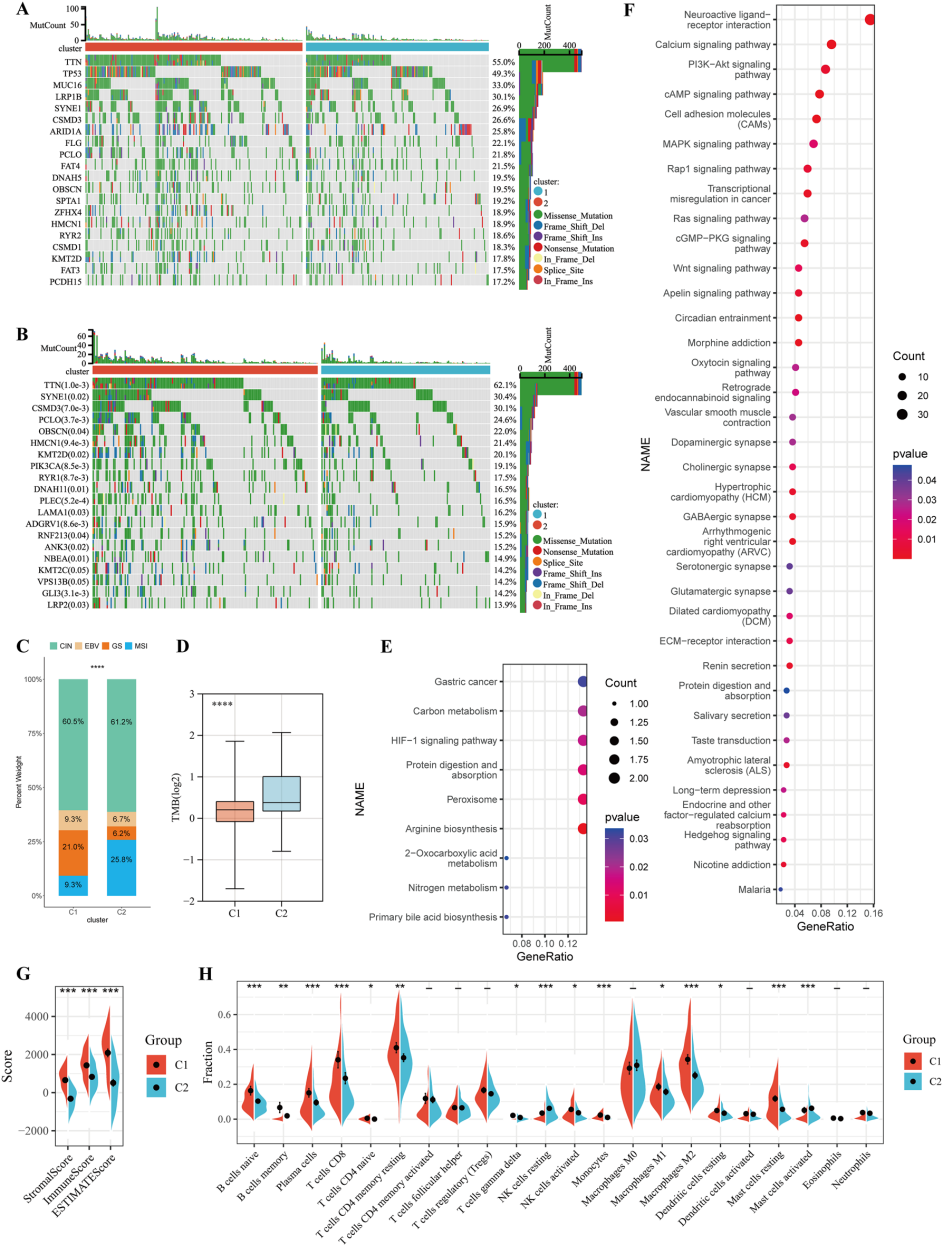
Differences between metabolic subtypes. (**A**) Top 20 mutated genes in all gastric cancer patients of TCGA-STAD cohort. (**B**) Top 20 differentially mutated genes in all gastric cancer patients of TCGA-STAD cohort. (**C**) Comparison of TCGA gastric cancer subtypes among two metabolic subtypes. (**D**) comparison of tumor mutation burden among two metabolic subtypes. (**E**) KEGG signaling pathway enriched for genes with low methylation and high expression in cluster 2. (**F**) KEGG signaling pathway enriched for genes with low methylation and high expression in cluster 1. (**G**) Stromal score, Immune score and ESTIMATE score between two metabolic subtypes. (**H**) Relative proportion of 22 infiltrating immune cells estimated by CIBERSORT between two metabolic subtypes of TCGA-STAD cohort. *P < 0.05, **P < 0.01, ***P < 0.001, ****P < 0.0001.

Oncogene mutations have been shown to induce reprogramming of cell-autonomous metabolism. To further investigate whether there is evidence of the disparity in the genomic layer between the two metabolic subtypes, we analyzed somatic mutations in the TCGA-STAD cohort. Top 20 most frequently mutated genes in two subtypes were illustrated in Fig. 3A. And we also analyzed genes that were differentially mutated in the two subtypes (Fig. 3B). The top 5 most frequently mutated genes in gastric cancer patients are TTN, TP53, MUC16, LRP1B and SYNE1 (Fig. 3A). Among them, TTN and SYNE1 are different between the two metabolic subtypes (Fig. 3B). The top 20 differentially mutated genes were more frequently mutated in the cluster 2, indicating that the formation of metabolic subtypes may be related to genes mutations. We found that chromosomal instability (CIN) and Epstein–Barr virus (EBV) subtypes had consistent distribution between cluster 1 and cluster 2, but cluster 1 had more genomically stable (GS) subtype, while cluster 2 had more microsatellite instability (MSI) subtype (Fig. 3C). We also found that cluster 2 subtype also had significantly higher tumor mutational burden (TMB) levels (Fig. 3D).

We removed CpG sites that have no corresponding gene, as well as the completely unmethylated or fully methylated CpG sites in both metabolic subtypes, leaving us with 71,648 CpG sites with P < 0.05. After applying a delaBeta of 0.15 to further screen for differential CpG sites, we identified 1431 hypomethylated genes in cluster 1 and 2410 hypomethylated genes in cluster 2. Among the hypomethylated genes in cluster 1, 480 genes were highly expressed, while among those in cluster 2, only 25 genes were highly expressed. The results of the enrichment analysis of these genes are shown in Fig. 3E,F. Interestingly, genes with hypomethylation and high expression in cluster 1 were enriched in oncogenic signaling pathways, suggesting that abnormal methylation may lead to the activation of oncogenic signaling pathways in cluster 1. Genes with hypomethylation and high expression in cluster 2 were enriched in HIF-1 signaling pathway, indicating that cluster 2 characteristics may be associated with hypoxia. Interactions between tumor cells and surrounding infiltrating cells, especially stromal cells and immune cells, can either promote or inhibit tumor progression^44^. We calculated the score of stromal and immune using ESTIMATE algorithm. The three scores in the cluster 1 group were significantly higher than those in the cluster 2 group (Fig. 3G), suggesting that the samples in cluster 1 had more non-tumor cell components. To further explore the differences of immune cell composition, CIBERSORT algorithm was implemented to assess the composition of 22 immune cells in the TCGA-STAD cohort (Fig. 3H). The samples by CIBERSORT that generated P-values greater than 0.05 were removed. Only 7 immune cells (T cells CD4 memory activated, T cells follicular helper, T cells regulatory (Tregs), Macrophages M0, Dendritic cells activated, Eosinophils and Neutrophils) did not differ between the two groups. All diverse immune signatures, except NK cells resting and Mast cells activated, were increased in the cluster 1 subgroup. The above results indicated that the tumor immune microenvironment may be associated with gastric cancer metabolic subtypes.

### Metabolic subtype-associated treatment strategy for gastric cancer

**Figure 4:**
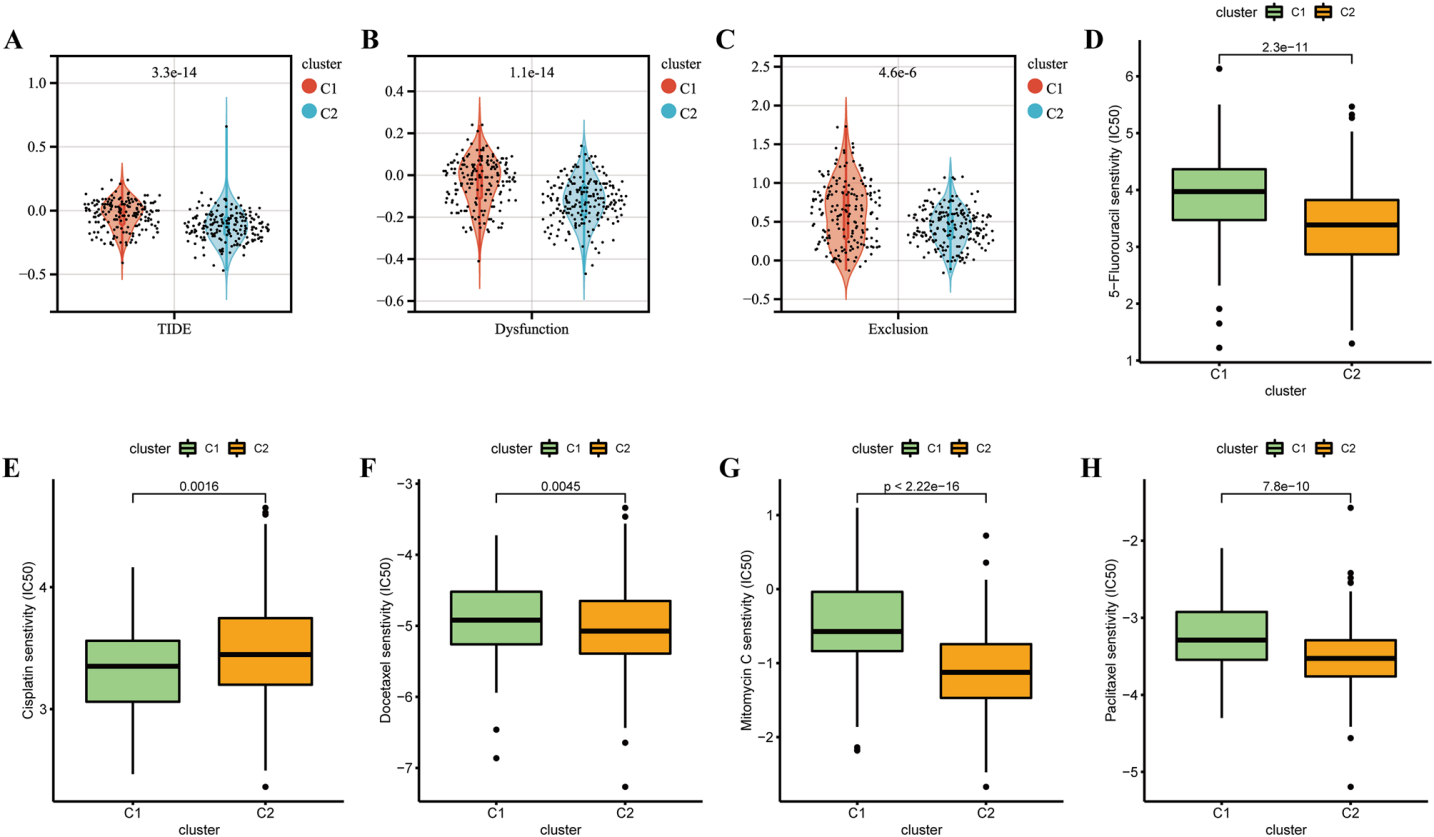
The estimation of immunotherapy response, chemotherapy response and potential therapeutic drugs for gastric cancer. (**A**) TIDE scores of two metabolic subtypes in the TCGA-STAD cohort. (**B**) T cell dysfunction scores of two metabolic subtypes in the TCGA-STAD cohort. (**C**) T cell exclusion scores of two metabolic subtypes in the TCGA-STAD cohort. (**D**–**H**) The chemotherapy response of two metabolic subtypes for 5 common chemotherapy drugs.

To assess the association of metabolic subtypes with immunotherapy, we adopted Tumor Immune Dysfunction and Exclusion (TIDE; http://tide.dfci.harvard.edu/) for TCGA-STAD cohort. The cluster 1 subgroup had higher TIDE scores, indicating that the cluster 1 subgroup had a higher probability of immune escape and thus lower benefit from immunotherapy (Fig. 4A). We also compared the n T cell dysfunction scores and T cell exclusion scores between the two subgroups, which supported the above conclusion (Fig. 4B,C). The above analysis suggested that cluster 2 subgroup may have a better response to immunotherapy.

The previous findings showed that metabolic subtypes were associated with drug metabolism signaling pathways, which led us to explore their potential as a marker for predicting drug response. The Cancer Genome Project (CGP) database was used to predict chemotherapeutic response. We found 5 drugs commonly used in gastric cancer chemotherapy in the CGP database, and the estimated IC50 of these 5 drugs were significantly differed between the two subgroups (Fig. 4D–H). The patients with metabolic subtype-2 were more sensitive to the anticancer drugs 5-fluorouraci, docetaxel, mitomycin C and paclitaxel. The patients with metabolic subtype-1 were more sensitive to cisplatin.

Furthermore, we screened the CMap database for small-molecule drugs with therapeutic effects on gastric cancer, based on differentially expressed genes between the two metabolic subtypes. As a result, we identified three potential small molecule drugs for gastric cancer (dimercaptosuccinic-acid, lapatinib, tracazolate).

### Metabolic subtype-associated signature is a prognostic indicator for gastric cancer

**Figure 5:**
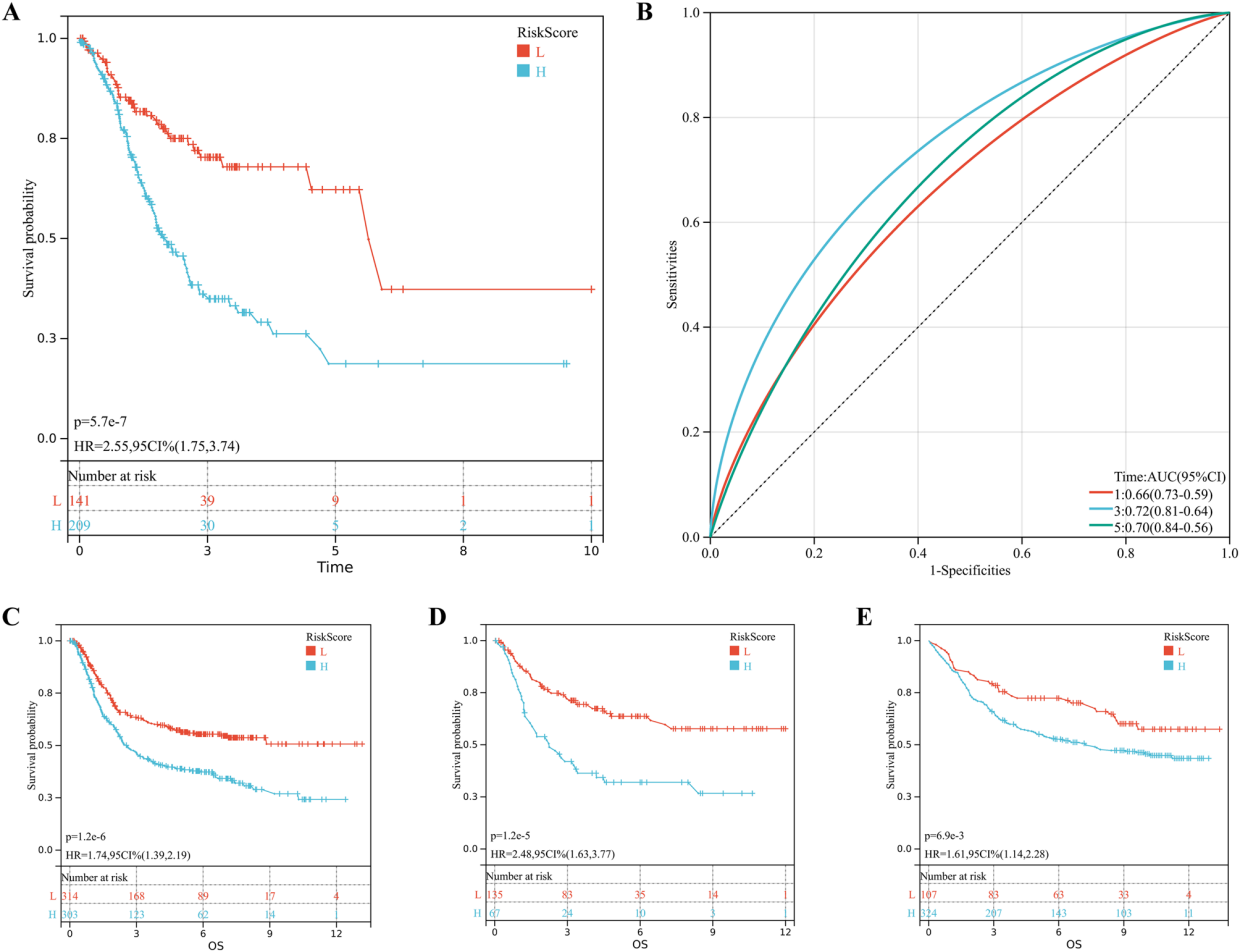
Construction of metabolic subtype specific prognostic model. (**A**,**B**) Patients were divided into high-risk and low-risk subgroup based best cutoff, Kaplan–Meier analysis demonstrated that patients with higher metabolic subtype-associated signature score exhibited worse overall survival in TCGA-STAD, ROC curves showing the predictive efficiency of the model on the 1-, 3-, and 5-years survival rate. (**C**–**E**) the prognostic difference was validated in 3 independent cohorts.

Multiple datasets demonstrated significant prognostic differences between two metabolic subtypes. Therefore, we explored whether a metabolic subtype-related signature could be used to predict patient outcomes. First, we performed differential analysis to obtain a list of differentially expressed genes between metabolic subtypes. Subsequently, LASSO Cox algorithm with 0.07 of the optimal λ value in the model was applied to identify the most robust prognostic genes based on differentially expressed genes profiles after Z-score transformed (Fig. S3A,B). KM analysis revealed that patients with a low metabolic subtype-associated signature score demonstrated a prominent survival benefit (log-rank test, p = 5.7e−7; Fig. 5A). Furthermore, ROC analysis also showed that this model can accurately predicted patient survival time (Fig. 5B). We also performed KM analysis on these 11 genes separately, and found that the expression levels of nine genes were associated with the prognosis of gastric cancer patients (Fig. S4A–K).

In order to validate the stability of the model, we performed analyses in three additional cohorts. The results revealed that the low metabolic subtype-associated signature score group still had better survival in the three cohorts (GPL570 meta-dataset: HR = 1.74, 95% CI = 1.39–2.19, p = 1.2e−6; GSE26942: HR = 2.48, 95% CI = 1.63–3.77, p = 1.2e−5; GSE84437: HR = 1.61, 95% CI = 1.14–2.28, p = 6.9e−3; Fig. 5E–G).

### Metabolic features of epithelial cells in the single-cell resolution

**Figure 6:**
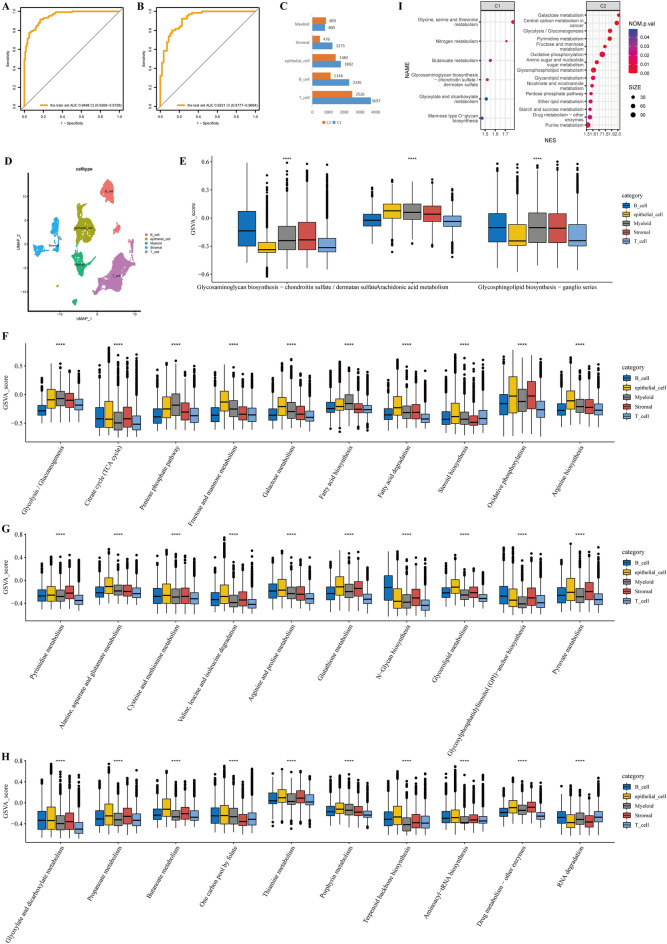
Analysis of metabolic subtypes in the single-cell dimension. (**A**,**B**) Performance of metabolic subtype classification models on training and test datasets. (**C**) Quantitative distribution of the five major cell types between the two metabolic subtypes. (**D**) The UMAP plot of all cells, which are color-coded based on their associated clusters. (**E**) Differences among the three metabolic pathways enriched in cluster 1 at the bulk dimension among the five types of cells. (**F**–**H**) Differences among the 30 metabolic pathways enriched in cluster 2 at the bulk dimension among the five types of cells. *P < 0.05, **P < 0.01, ***P < 0.001, ****P < 0.0001.

Tumor tissue contains a variety of non-tumor cells that play an important role. Therefore, we aimed to explore the differences between metabolic subtypes at a single-cell resolution. Among the eight gastric cancer samples analyzed, four were intestinal-type, two were diffuse-type, and one was mixed-type (Table S3). After quality control processing, there were 16,397 cells left, and we normalized the count data using “LogNormalize” method built into "Seurat" package. Subsequently, we utilized a classification model to perform metabolic classification of patients with single-cell sequencing. First, we calculated a matrix of average expression values of all genes in all cells of a single patient. We divided TCGA-STAD into training set and test set, and the SVM classification model showed extremely high accuracy in both training set and test set (the train set: AUC = 0.9498, 95% CI = 0.9268–0.9728; the test set: AUC = 0.9231, 95% CI = 0.8777–0.9684; Fig. 6A,B). We then classified the 8 patients into 2 metabolic subgroups according to this classification model (details in Table S4). The expression data of the above SVM analysis have been transformed by Z-score. The ssGSEA algorithm was used to assess the relevant KEGG signaling pathway levels in 8 single-cell sequencing samples. The results were the same to those obtained from bulk sequencing, with the high-level pathways in bulk cluster 1 subtype activated in single-cell cluster 1 subtype (Fig. S5A), and similarly for the pathways enriched in cluster 2 subtypes (Fig. S5B). Although the p-value was insignificant due to the small sample size, there was a clear trend in the results from Fig. S5A,B, indicating the robustness of the single-cell sample classification.

All cells were annotated into 5 cell types based on relevant markers (detailed markers in Table S5, Fig. 6D), including epithelial cell, T cell, B cell, stromal cell and myeloid cell. The numbers of 5 cells in the two metabolic subtypes are shown in Fig. 6C. The ssGSEA algorithm was used to assess the relevant KEGG signaling pathway levels in 16,397 cells. We wondered whether epithelial cells dominate the metabolic signaling pathways associated with metabolic subtypes. Three metabolic signaling pathways were mainly enriched in metabolic subtype 1, but only one was dominated by epithelial cells (Fig. 6E). Interestingly, the majority of the 30 metabolic signaling pathways enriched in metabolic subtype 2 were still dominated by epithelial cells (Fig. 6F–H).

To make the results more credible, we screened 1144 malignant epithelial cells from epithelial cells using the “CopyKAT” algorithm. GSEA analysis was used to analyze the metabolic heterogeneity of various cells in different metabolic subtypes. Six metabolic pathways were enriched in malignant epithelial cells of metabolic subtype 1, including the Glycosaminoglycan biosynthesis—chondroitin sulfate/dermatan sulfate pathway, which was consistent with bulk sequencing analysis (Fig. 6I). Then, 15 metabolic pathways were enriched in malignant epithelial cells of metabolic subtype 2, of which 8 were also enriched in subtype 1 of the bulk data (Fig. 6I). In addition, we analyzed the metabolic pathways of normal epithelial cells and malignant epithelial cells. The results indicated that normal epithelial cells did not show any significant enrichment of metabolic pathways, while malignant epithelial cells exhibited activation of 57 metabolic pathways (Table S6). Taken together, our findings highlight the metabolic heterogeneity of malignant cells.

### Metabolic features of non-epithelial cells in the single-cell resolution

**Figure 7:**
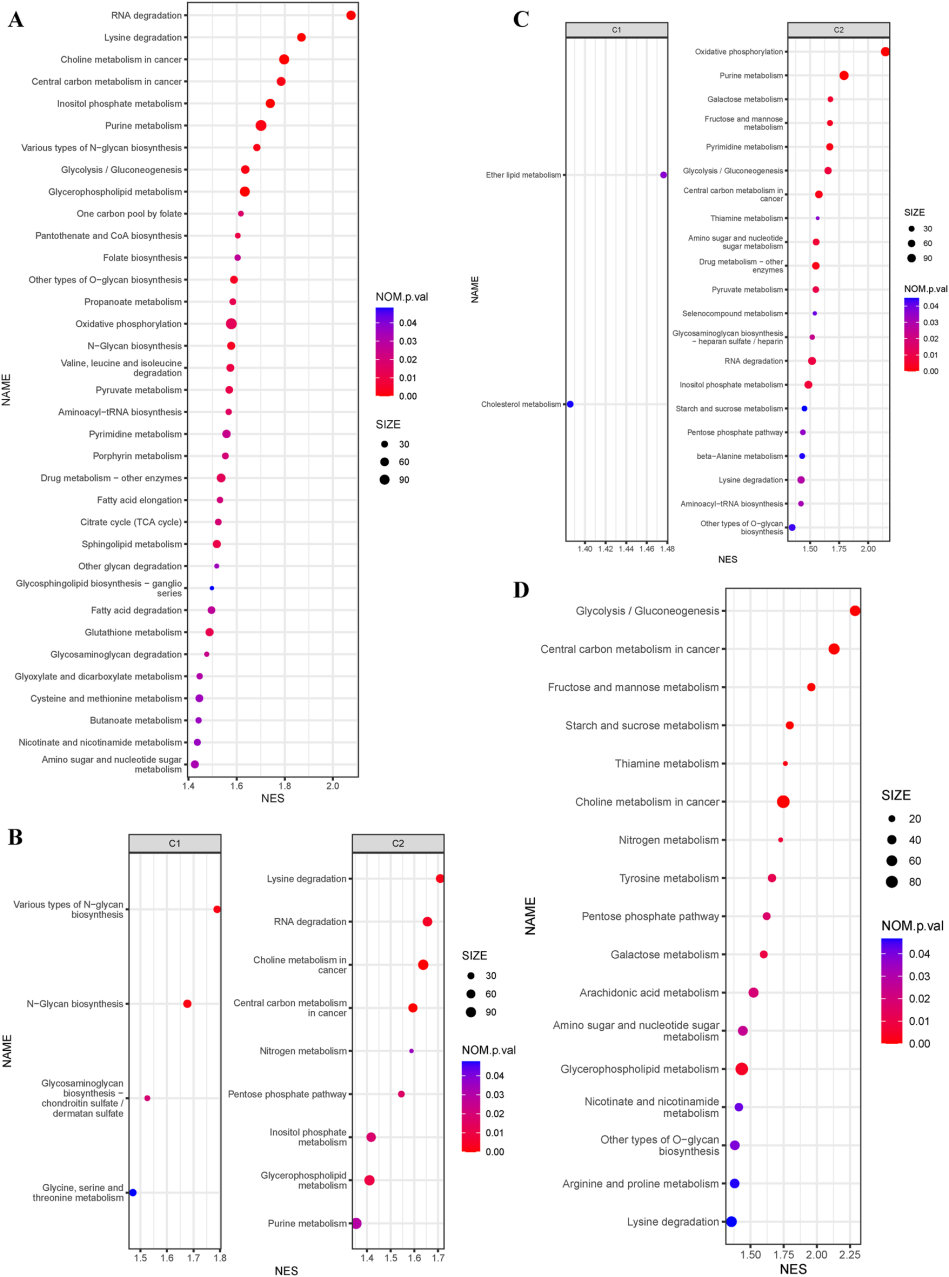
Metabolic pathway analysis of non-epithelial cells in single-cell sequencing. (**A**) Metabolic pathways analysis in T cells of different metabolic subtypes using Gene set enrichment analysis (Only metabolic subtype 2 had significantly enriched metabolic pathways). (**B**) Metabolic pathways analysis in B cells of different metabolic subtypes using Gene set enrichment analysis. (**C**) Metabolic pathways analysis in stromal cells of different metabolic subtypes using Gene set enrichment analysis. (**D**) Metabolic pathways analysis in myeloid cells of different metabolic subtypes using Gene set enrichment analysis (only metabolic subtype 2 had significantly enriched metabolic pathways).

The non-epithelial cells were classified into 2 metabolic subtypes, based on their metabolic pathways. The four types of non-epithelial cells have specific metabolic pathways and common metabolic pathways (Fig. 7A–D). Metabolic subtype 2 showed stable activation of central carbon metabolism in cancer pathway and lysine degradation pathway in non-epithelial cells. On the other hand, only a small number of metabolic pathways were significantly enriched in metabolic subtype 1, specifically in B cells and stromal cells (Fig. 7B,C). These findings indicate that not only epithelial cells but also all non-epithelial cells exhibit metabolic characteristics of metabolic subtype 2, which is abundant in nature.

## Discussion

Due to the heterogeneity of gastric cancer, the existing treatment methods are inevitably ineffective for some patients. Therefore, it is urgent to classify gastric cancer patients based on the existing data to discover potential subtypes of gastric cancer and facilitate personalized treatment. Metabolic reprogramming of tumor cells is required for tumorigenesis and progression. Tumor cells autonomously alter their phenotype through various metabolic pathways to meet increased energetic and biosynthetic demands. Therefore, we classified gastric cancer patients into two subtypes based on metabolic gene expression and conducted a detailed analysis of their differences from the genomic, epigenetic, and single-cell dimensions.

Our analysis classified gastric cancer patients into two metabolic subgroups, with cluster 1 consistently indicating a worse prognosis. We found that metabolic subtype cluster 1 was characterized by low metabolism, while cluster 2 was characterized by abundant and higher metabolism. Pentose phosphate pathway, Glycolysis/Gluconeogenesis pathway and Citrate cycle (TCA cycle) maintained high levels in cluster 2. In addition, cluster 2 was enriched with multiple amino acid metabolism-related pathways, which also indicated the high metabolic feature of cluster 2. Analysis of 5 tumor metabolism-related pathways may partially explain of the metabolic differences between the two clustered subtypes^45–49^. More of the 10 tumor-related pathways were highly activated in cluster 1, indicating that cluster 1 had a higher degree of malignancy and therefore, a shorter survival time.

After conducting genomic analysis, we observed that cluster 2 exhibited a higher incidence of gene mutations, which is consistent with the above-mentioned activation of several nucleotide processing and repair-related pathways in cluster 2. Epigenetic analysis showed that the hypomethylated genes in cluster 1 were mostly oncogenic signaling pathways-related genes, leading us to believe that the high malignancy of cluster 1 may be related to gene hypomethylation. Both CIBERSORT and ESTIMATE analyses demonstrated that cluster 1 possessed a more abundant immune cell infiltration. Although cluster 1 had more abundant immune cell infiltration, we found that naive cells and memory cells, as well as various immune cells with immunosuppressive effects, were more abundant in cluster 1. However, the activated cells that exert anti-tumor effects did not differ between the two groups. This may explain high immune cell infiltration in cluster 1 but shorter survival time.

Given the notable prognostic differences between the two metabolic subtypes, we explored subtype-specific genes as potential prognostic markers. The results were also satisfactory, with the 6-gene prognostic model not only showing satisfactory results in the TCGA-STAD cohort, but also performing extremely well in three other independent data cohorts.

Based on the above two metabolic subtypes, we explored and discovered different treatment strategies. We predicted the therapeutic effects of 5 common chemotherapeutic agents in different metabolic subtypes. According to IC50 estimates, cluster 1 patients were more sensitive to cisplatin, while cluster 2 was more sensitive to 5-fluorouraci, docetaxel, mitomycin C and paclitaxel. This information enables medical professionals to more precisely select a suitable chemotherapy program for their patients. TIDE analysis suggested that cluster 2 patients may benefit more from immunotherapy than cluster 1 patients.

In addition, we further explored the metabolic differences of various cells at the single-cell level, focusing on different metabolic subtypes. In most subtype-related metabolic pathways, most of them are dominated by epithelial cells. To enhance the validity of our findings, we also separated from malignant epithelial cells from epithelial cells, as a result, the malignant epithelial cell metabolism is higher. Then, five major types of cells (epithelial cells, T cells, B cells, stromal cells and myeloid cells) analysis indicate that various cells in cluster 2 are in high metabolic level, and the cells in cluster 1 are in a relatively low metabolic level. Therefore, the cluster 1 subtype is metabolically indolent gastric cancer. The low-metabolic level of immune cells in cluster 1 may be associated with its poor prognosis and low immunotherapy response.

Nonetheless, our research has some limitations. Specifically, we did not investigate metabolic subtypes at the protein level, which could be an area for future research.

## Conclusion

We provide a new perspective on the heterogeneity of gastric cancer from the metabolic. And we reveal the characteristics of metabolic subtypes from the genome, DNA methylation and single cells.

## Supplementary Information

Supplementary Figures.

Supplementary Tables.

## Data Availability

The data that support the findings of this study are available in GEO (https://www.ncbi.nlm.nih.gov/geo/, GSE62254, GSE15459, GSE57303, GSE34942, GSE84437, GSE26942 and GSE183904), TCGA (https://portal.gdc.cancer.gov/repository, TCGA-STAD), and the Supporting Information.

## Abbreviations

GC: Gastric cancer
ICIs: Immune checkpoints targeting immunotherapies
GEO: Gene-Expression Omnibus
TCGA: The Cancer Genome Atlas
TPM: Transcripts per million
DMPs: Differentially methylated probes
SVM: Support vector machines
KEGG: Kyoto Encyclopedia of Genes and Genomes
GSEA: Gene set enrichment analysis
ssGSEA: Single sample gene set enrichment analysis
FDR: False discovery rate
KM: Kaplan–Meier
ROC: Receiver operating characteristic
AUC: The area under the curve
IC50: The half maximal inhibitory concentration
OS: Overall survival
TIDE: Tumor Immune Dysfunction and Exclusion
CGP: The Cancer Genome Project
