# Supplementary material for: Bulk and single-cell transcriptome profiling reveal the metabolic heterogeneity in gastric cancer
Source: Sci Rep. 2023 May 31;13:8787. doi: 10.1038/s41598-023-35395-y (PMC10232450; doi:10.1038/s41598-023-35395-y)
Supplement: Supplementary file 1 — Supplementary Figures. [file 41598_2023_35395_MOESM1_ESM.docx]

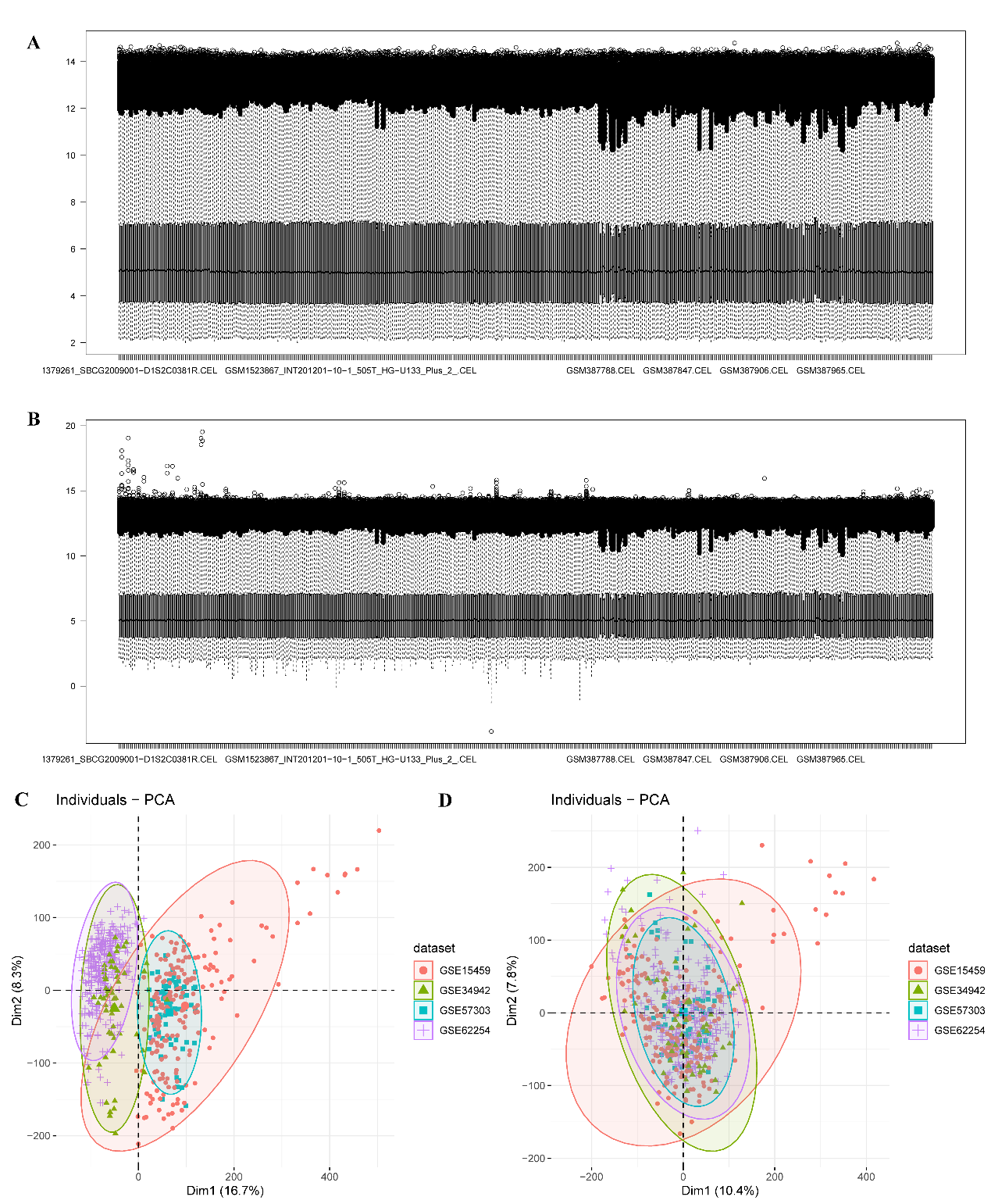


Figure S1. (**A**) Boxplot before batch effect correction. (**B**) Boxplot after batch effect correction. (**C**) PCA plot before batch effect correction. (**D**) PCA plot after batch effect correction. (We used the oligo package in R software for quality control analysis.)


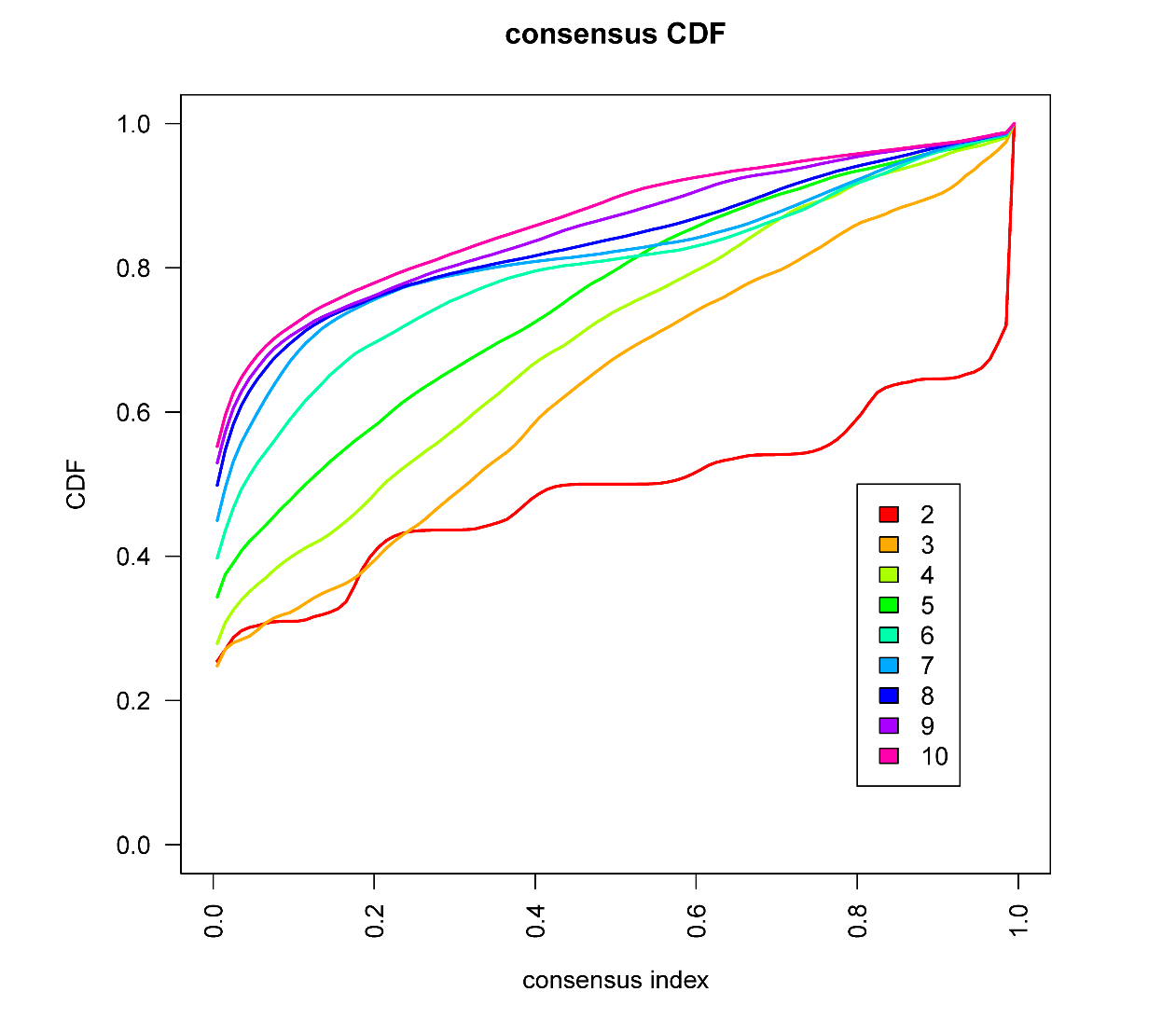


Figure S2. The curve of K = 2 in the CDF plot has the smallest change value.


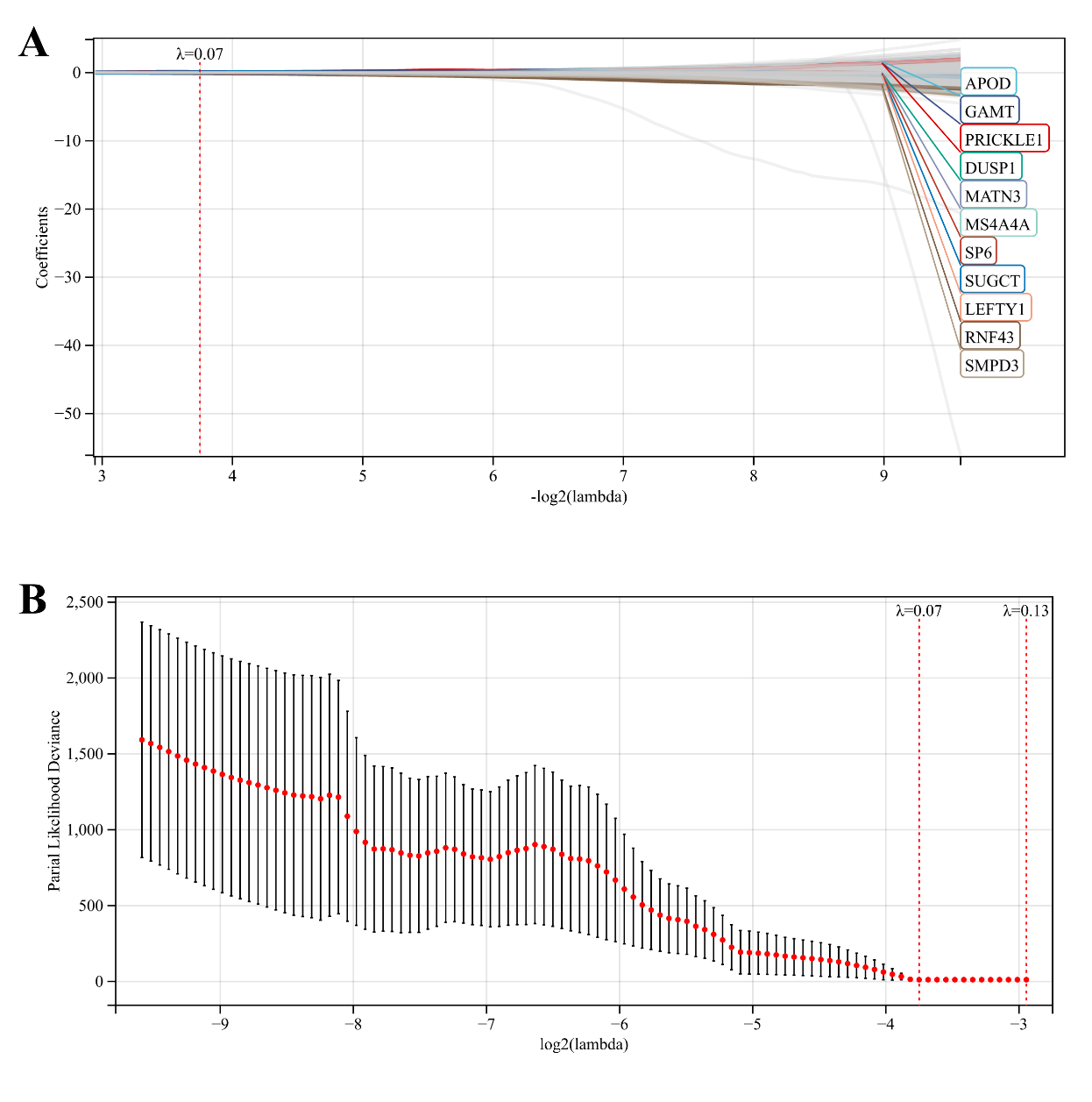


Figure S3. Construction of metabolic subtype specific prognostic model. (**A and B**) Partial likelihood deviance for the lasso regression and Lasso regression analysis.


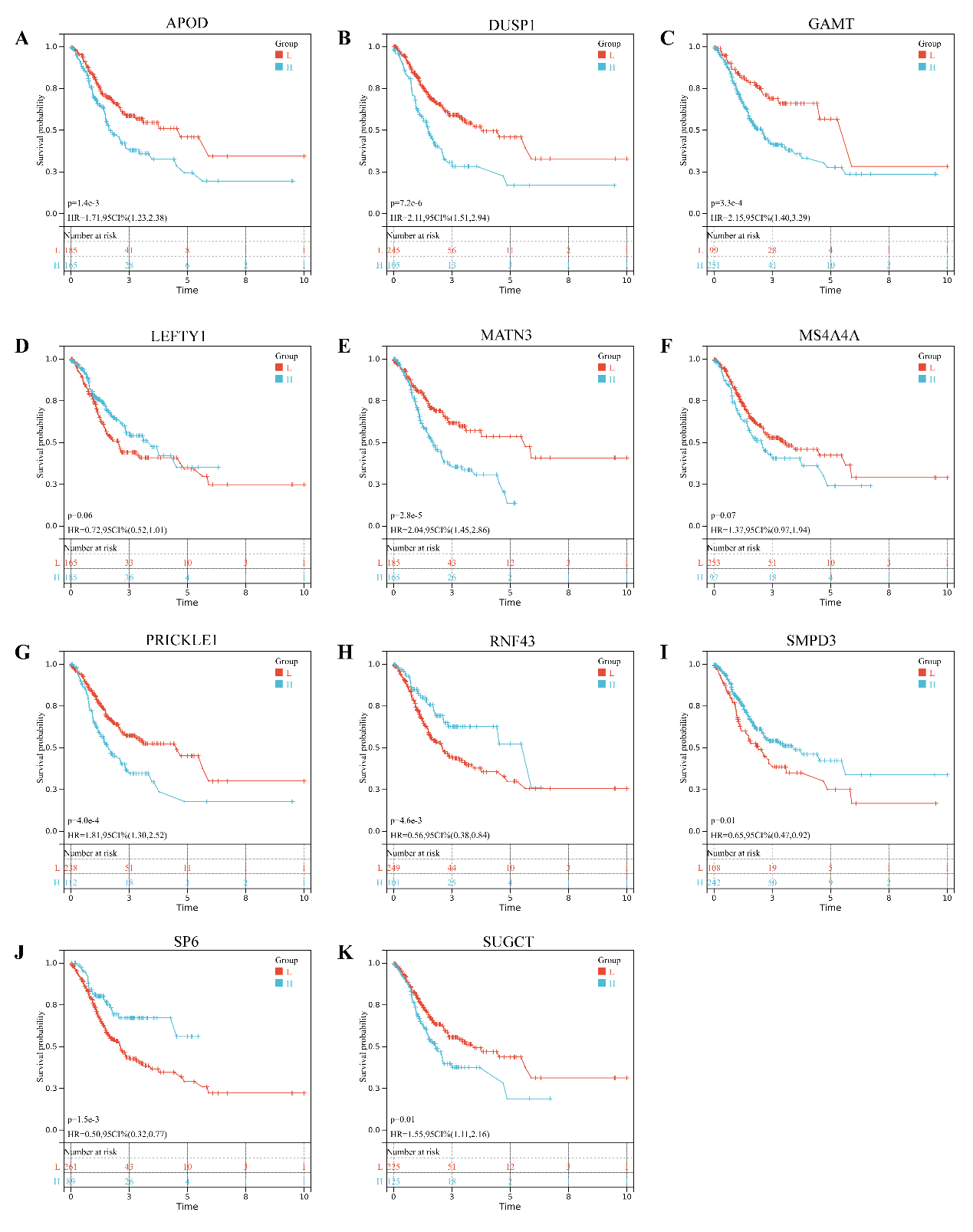


Figure S4. (**A-K**) Kaplan-Meier analysis of 6 marker genes related to metabolic subtype.


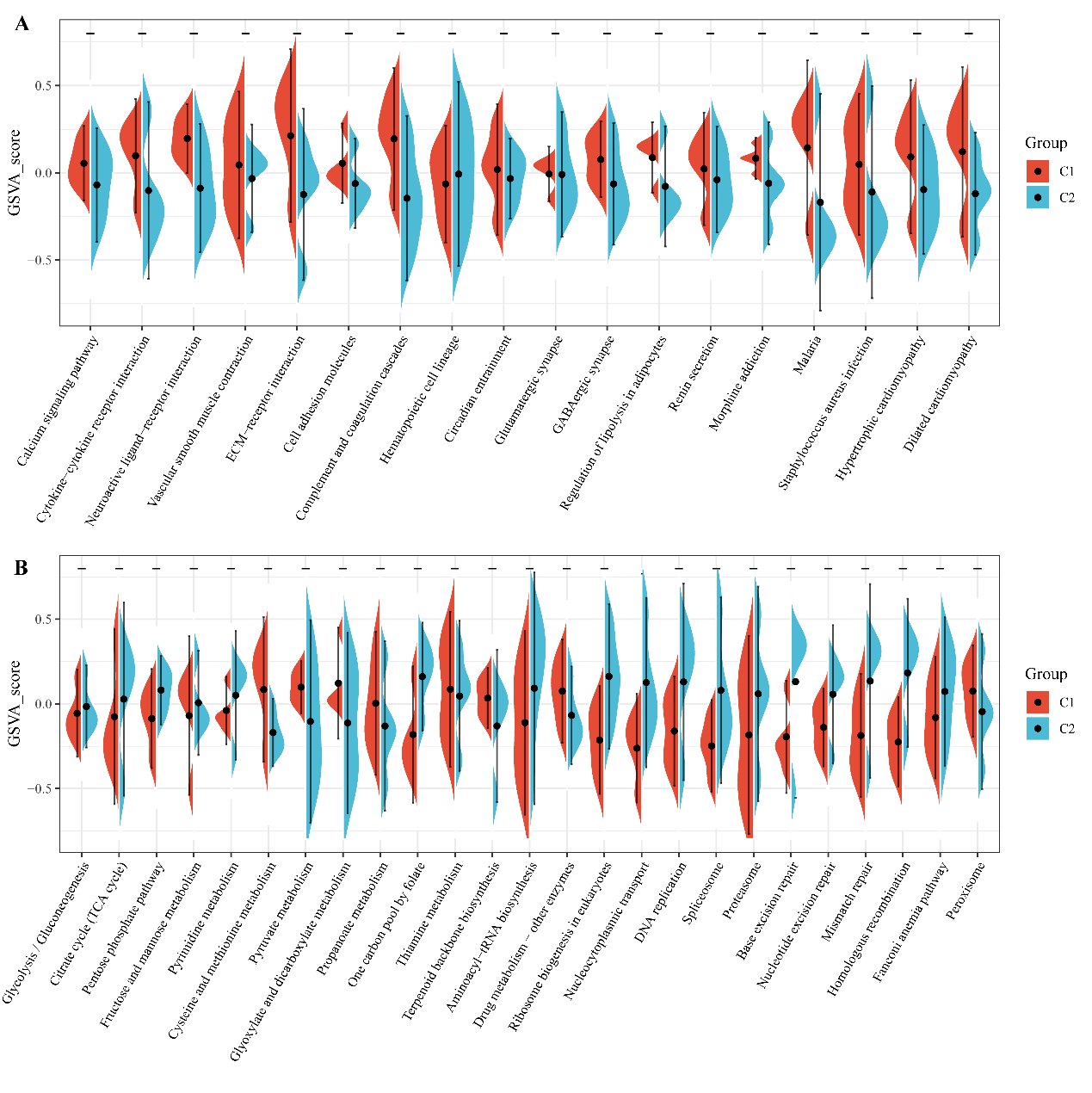


Figure S5. (**A**) The high-level pathways in bulk cluster 1 subtype were still activated in single-cell cluster 1 subtype. (**B**) The high-level pathways in bulk cluster 2 subtype were still activated in single-cell cluster 2 subtype.
